# Identification of a lncRNA based signature for pancreatic cancer survival to predict immune landscape and potential therapeutic drugs

**DOI:** 10.3389/fgene.2022.973444

**Published:** 2022-09-14

**Authors:** Di Ma, Yuchen Yang, Qiang Cai, Feng Ye, Xiaxing Deng, Baiyong Shen

**Affiliations:** ^1^ Department of General Surgery, Ruijin Hospital, Shanghai Jiao Tong University School of Medicine, Shanghai, China; ^2^ Shanghai Key Laboratory of Translational Reseach for Pancreatic Neoplasms, Research Institute of Pancreatic Diseases, Shanghai Jiao Tong University School of Medicine, Shanghai, China

**Keywords:** drug response, immune landscape, prognostic model, long non-coding RNA, pancreatic cancer

## Abstract

Pancreatic cancer is one major digestive malignancy with a poor prognosis. Given the clinical importance of lncRNAs, developing a novel molecular panel with lncRNAs for pancreatic cancer has great potential. As a result, an 8-lncRNA-based robust prognostic signature was constructed using a random survival forest model after examing the expression profile and prognostic significance of lncRNAs in the PAAD cohort from TCGA. The efficacy and effectiveness of the lncRNA-based signature were thoroughly assessed. Patients with high- and low-risk defined by the signature underwent significantly distinct OS expectancy. Most crucially the training group’s AUCs of ROC approached 0.90 and the testing group similarly had the AUCs above 0.86. The lncRNA-based signature was shown to behave as a prognostic indicator of pancreatic cancer, either alone or simultaneously with other factors, after combined analysis with other clinical-pathological factors in Cox regression and nomogram. Additionally, using GSEA and CIBERSORT scoring methods, the immune landscape and variations in biological processes between high- and low-risk subgroups were investigated. Last but not least, drug databases were searched for prospective therapeutic molecules targeting high-risk patients. The most promising compound were Afatinib, LY-303511, and RO-90-7501 as a result. In conclusion, we developed a novel lncRNA based prognostic signature with high efficacy to stratify high-risk pancreatic cancer patients and screened prospective responsive drugs for targeting strategy.

## Introduction

Pancreatic cancer is known as a highly lethal malignancy with a poor prognosis. Accounting for approximately 496,000 new patients per year worldwide, pancreatic cancer ranks 14th in new cases among 36 major types of cancer (Sung et al., 2021). However, the disease reaches as high as the 4th in cancer-related death, with the death number around 466,000 annually. On average, the 5-year survival rate of pancreatic cancer is below 10% at the time of diagnosis (Mizrahi et al., 2020; Zhu et al., 2021). Nowadays, surgical resection remains the optimal treatment for pancreatic cancer by increasing the 5-year survival rate to around 20% (Christenson et al., 2020). For unresectable tumors, nonetheless, the efficacy of other approaches such as chemotherapy, radiotherapy, and systemic therapies, despite receiving incremental progress during the last decade, requires further assessment. To note, due to lacking severe symptoms in the early stage, the diagnosis of pancreatic cancer is of great difficulty and often delayed. Therefore, novel molecular markers of satisfying sensitivity and accuracy become an urgent demand for diagnosis and prognosis evaluation purposes. Besides, new strategies for screening patients with higher long-term risk are also expected for better clinical decision-making.

Not surprisingly, considerable amounts of studies have revealed the possibility and value of molecular signatures in the diagnosis and prognosis of pancreatic cancer over the last few years. For instance, Wu et al. have developed a nine-gene (MET, KLK10, COL17A1, CEP55, ANKRD22, ITGB6, ARNTL2, MCOLN3, and SLC25A45) panel to predict the overall survival of pancreatic cancer (Wu et al., 2019). Other groups have also built several signatures based on genes associated with different biological aspects of pancreatic cancer including autophagy, methylation, and metabolic changes (Yu et al., 2021; Xiao et al., 2022; Zhang et al., 2022). Nowadays it is widely acknowledged that the dysregulation of non-coding RNA is closely correlated to different types of tumors including pancreatic cancer. Hence, signature classifiers generated from non-coding RNAs have also been carried out using micro RNA (miRNA), long non-coding RNA (lncRNA), circular RNA (circRNA) and so forth. Nevertheless, the value of non-coding RNAs in assessing pancreatic cancer has not been thoroughly explored, as most of them emphasized prognosis prediction but failed to provide detailed hints on clinical decision making.

Therefore, this study aims to identify the clinical significance of lncRNAs for pancreatic cancer evaluation and construct a comprehensive lncRNA-based signature with high prognostic efficacy to monitor outcomes of pancreatic cancer patients. Besides, the molecular signature is used to explore the immune landscape and potential therapeutic targets and small molecules between risk subgroups. In detail, expression and clinical data of pancreatic cancer patients were acquired from public databases including The Cancer Genome Atlas (TCGA), Cbioportal, and Cancer Cell Line Encyclopedia (CCLE). An 8-lncRNA classifier was then constructed by applying Cox and random survival forest (RSF) regression in differentially expressed lncRNAs (DElncRNAs). The capacity of the signature as a prognostic indicator was evaluated in different aspects. To emphasize, the immune feature landscape, possible therapeutic targets, and molecules were subsequently checked in patients with a high-risk score according to the signature in detail, holding the potential to expand the current therapeutic strategies for the pancreatic cancer population.

## Materials and methods

### RNA-sequencing cohorts

An RNA-seq dataset of 177 pancreatic cancer patients involving RNA expression value and matched clinical information was obtained from the TCGA data portal (http://portal.gdc.cacner.gov/repository) and the Cbioportal website (http://cbioportal.org). Fragments per million reads (FPKM) normalized expression value was used for further analysis. The cohort was then randomly split with a 2:1 ratio into a training group and a testing group.

### Cancer cell line data

Expression profiles of human cancer cell lines (CCLs) were achieved from the Broad Institute CCLE project (http://portals.broadinstitute.org/ccle). To search for potential therapeutic agents, sensitivity data of compounds in CCLs were achieved from the Cancer Therapeutics Response Portal, Board institute (CTRP, http://portals.broadinstitute.org/ctrp) and PRISM repurposing dataset (http://depmap.org/portal/prism). The algorism of drug sensitivity was described in previous studies. Briefly, the database provided the area under the curve (AUC) values as the readout of drug sensitivity. The lower AUC values indicate higher drug sensitivity. Compounds with more than 20% missing data were excluded from the dataset, and the K-nearest neighbor algorithm (K-NN) was applied to estimate the AUC values. To further investigate the mechanism of actions (MoA) of the drugs screened out, the Connectivity Map tools database (CMap, http://clue.io) with more than 2000 small molecule perturbagen types was applied for specific analysis.

### Construction of lncRNA-based prognostic signature

The human lncRNA annotation profile was obtained from the GENCODE website (GRCh38.p13, release 39, http://gencodegenes.org/human). After the acquisition of lncRNA expression data as described above, the lncRNAs were separated from gene-coding RNA and other non-coding RNAs. Differentially expressed lncRNAs (DElncRNAs) were identified with the criteria of absolute log2 fold-change (log2FC) > 1 and adjusted *p*-value < 0.05 between tumor and control tissues. Afterward, univariate Cox regression was conducted to identify prognostic lncRNAs which shared a correlation with the overall survival (OS) time of the patients in the cohort (*p* < 0.05). Thus, the candidate prognostic lncRNAs were determined by overlapping the DElncRNAs and prognostic lncRNAs.

To develop the lncRNA-based signature, the univariate Cox proportional hazards regression was first applied to preliminarily narrow the candidates using the training group. Subsequently, an RSF regression based on minimal depth was used to finally identify the signature. The RSF regression model underwent iteration 1,000 times to construct a lncRNA-based OS classifier with the largest C-index value. Eventually, multivariate Cox regression was employed to select candidates as independent indicators to form the 8-lncRNA-based signature retained for the next analysis. According to the classifier, each sample in the cohort was endowed with a risk score following the equation:

$$RiskScore=\overset{n}{\underset{k=1}{\sum }}Coef_{k}\times Exp_{k}$$

in which *Coef*_*k*_ was the coefficients of each sample, *Exp*_*k*_ was the expression of member lncRNAs of the signature. The cohort was divided into high-risk and low-risk groups by the mean value of the risk score. Afterward, the efficacy and effectiveness of the lncRNA classifiers in both training, and validation cohorts were evaluated by the Kaplan-Meier long-rank test, Time-dependent ROC curve analysis, multivariate Cox regression and nomogram scoring.

### Immune function analysis

Algorithms including CIBERSORT and ssGSEA were applied to compare the pattern of immune infiltration between high-risk and low-risk groups. Moreover, the Tumor Immune Dysfunction and Exclusion (TIDE) scoring method were employed to assess the response to immunotherapy, extent of immune dysfunction, immune exclusion, and microsatellite instability (MSI) for patients in high-risk and low-risk groups.

### Statistical analysis

All statistical analyses were conducted with the R software platform (v4.0.2, R Foundation for Statistical Computing, Vienna, Austria). Some major R packages included “edgeR,” “limma,” “survival,” “ROCR,” “ggplot2,” “pRRophetic,” and “randomForestSRC”. To compare variables in multiple groups, Student’s t-test and ANOVA analysis were used for parametric factors, whereas the Wilcoxon rank-sum test and Kruskal–Wallis test were applied for nonparametric factors. To measure the correlation of different variables, Spearman’s rank-order correlation and Pearson’s r correlation were set. Furthermore, Kaplan-Meier and the log-rank test were used for survival analysis. The area under the curve (AUC) was measured to judge the efficacy of the receiver operating characteristic curve. For all statistical calculations, a two-tailed *p* < 0.05 was considered significant.

## Results

### Construction of lncRNA based prognostic signature

**FIGURE 1:**
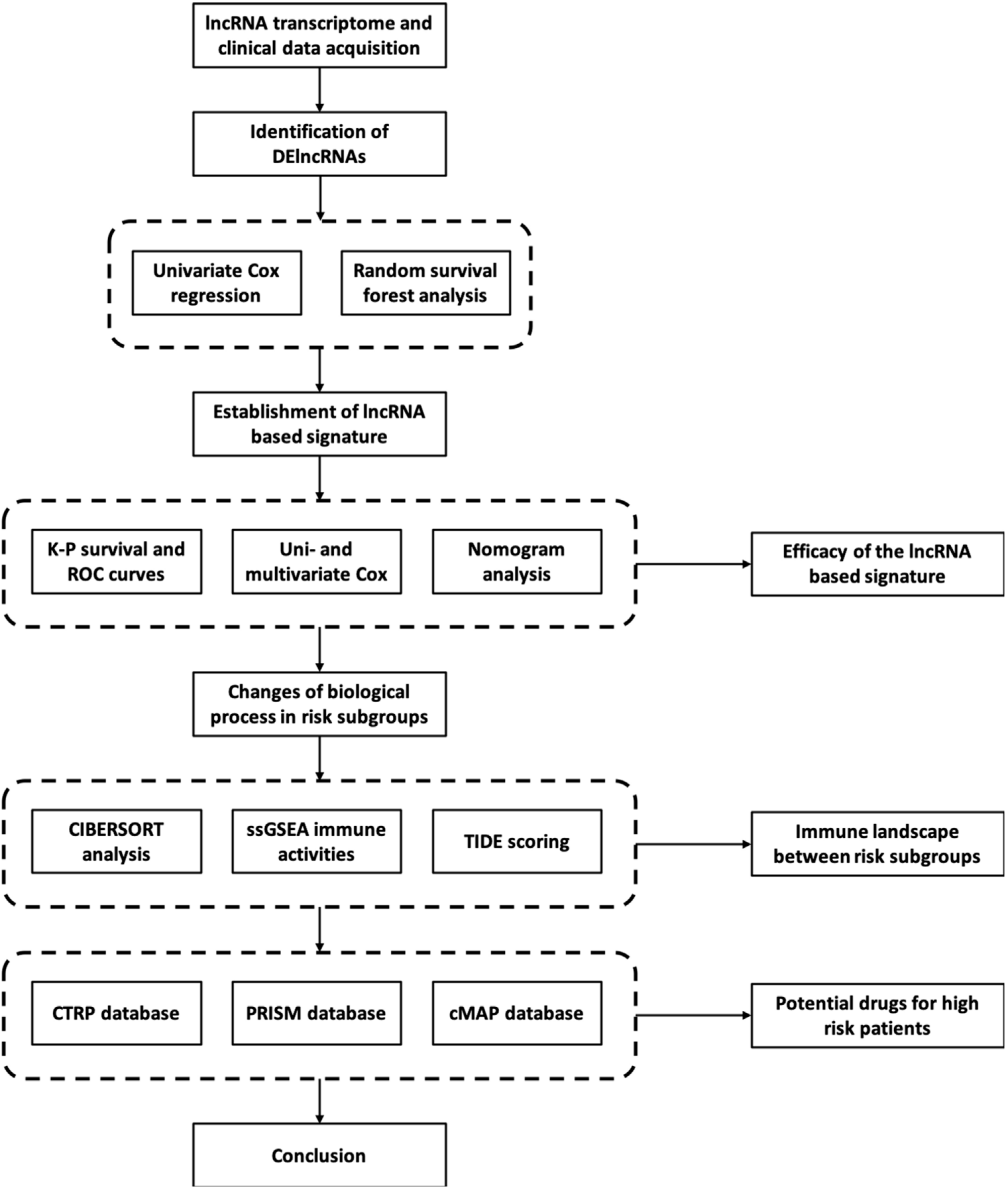
The work flow of the study.

**FIGURE 2:**
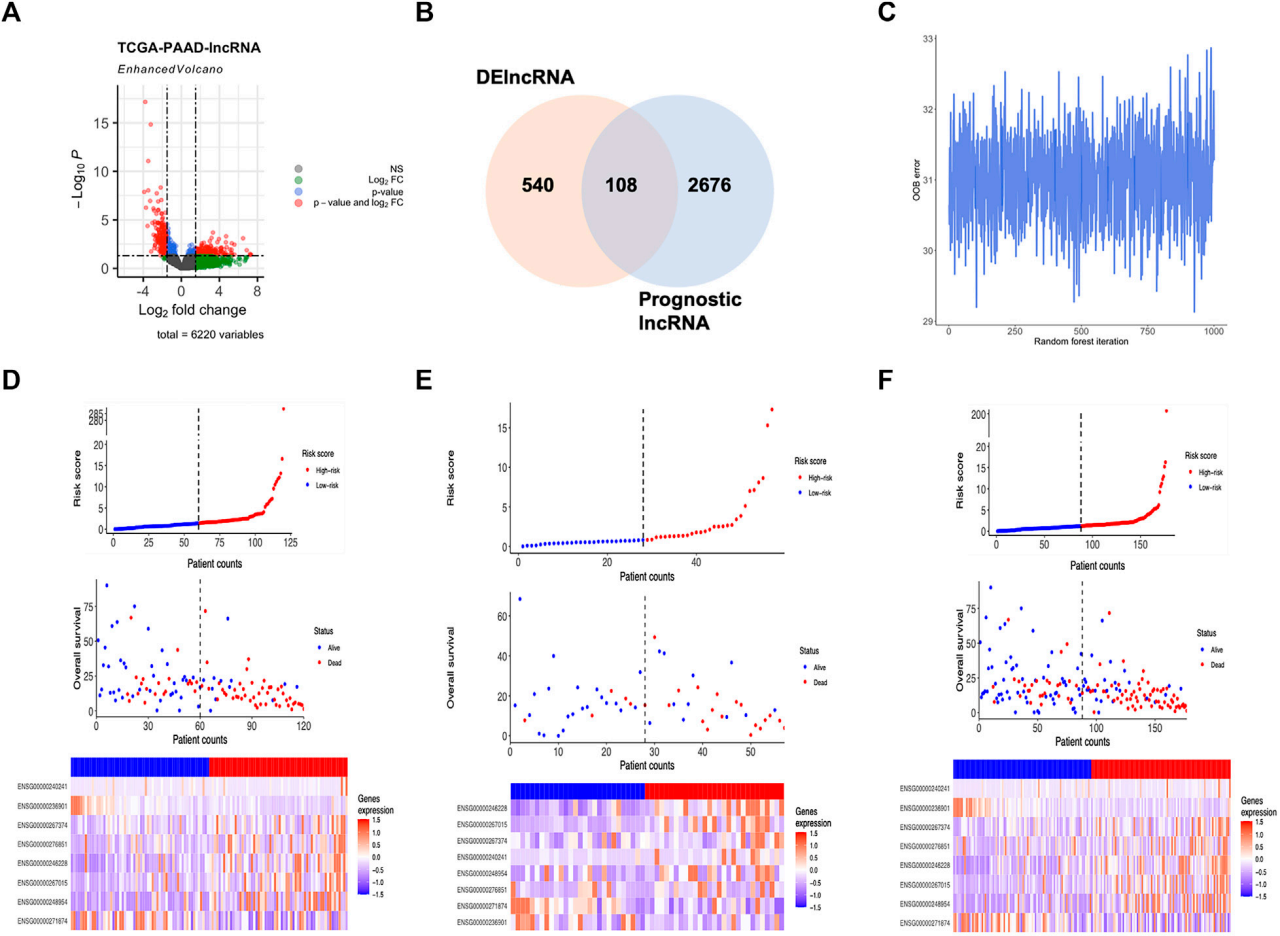
Development of lncRNA based molecular signature. **(A)** Volcano plot showed DElncRNAs identified from the TCGA-PAAD dataset. **(B)** Venn diagram of candidate lncRNAs obtained by overlapping DElncRNAs and Cox positive lncRNAs in the training cohort. **(C)** OOB error in 1,000 iteration of the random survival forest regression. **(D)** Distribution of the lncRNA based signature and expression of component lncRNAs in training group. **(E)** Distribution of the lncRNA based signature and expression of component lncRNAs in testing group. **(F)** Distribution of the lncRNA based signature and expression of component lncRNAs in whole group.

The whole RNA transcriptome profile containing tumor tissue (*n* = 177) and adjacent control (*n* = 4) was obtained from the TCGA portal as described above. Of the 14,078 lncRNAs extracted from the RNA-seq dataset, 540 lncRNAs were identified as DElncRNA under the condition of absolute log2 FC > 1 and adjust *p* < 0.05 (Figure 1; Figure 2A; Supplementary Figure S1). On the other hand, a univariate Cox regression analysis was used to select 2676 prognostic significant lncRNAs (*p* < 0.05) that possibly correlated to the OS time of the patients. A Venn diagram was created by overlapping DElncRNAs and univariate COX positive lncRNAs to select the candidate lncRNAs (Figure 2B). Next, a random survival forest (RSF) model was built based on minimal depth to screen out lncRNAs were most relevant to the prognosis. The RSF went through 1,000 times iterations under the criterion of largest C-index value and eventually led to an 8-lncRNA signature as the prognostic signature model for pancreatic cancer (Figure 2C). Thus, the multivariate Cox regression was applied to determine the risk score for each patient by values of coefficient and expression as described above (Supplementary Figure S2A). The detailed information on the elemental lncRNAs was listed as follows (Table 1).

**TABLE 1:** Information of the lncRNAs in pancreatic cancer prognostic signature. 8 lncRNA-based molecular classifier.

Gene id	Gene name	Chromosome	Start point	End point
ENSG00000236901	MIR600HG	9q33.3	125871773	125877756
ENSG00000267015	LncPQLC1-10	18q23	79337837	79344139
ENSG00000246228	CASC8	8q24.21	127277047	127482140
ENSG00000248954	Lnc-KAT7-3	17q21.33	49887597	49936831
ENSG00000271874	Lnc-RAD1-3	5p13.2	34647370	34656270
ENSG00000240241	Lnc-ROBO2-3	3p12.3	78266893	78298888
ENSG00000267374	MIR924HG	16q22.1	39113067	*39800322*
ENSG00000276851	Lnc-PDK2-5	17q21.33	50094064	50094647

### Assessment of the prognostic potentiality of lncRNA based signature

As the 8-lncRNAs-based classifier for pancreatic cancer was constructed, its efficacy for prognosis induction was evaluated in all aspects. First, patients were divided into high-risk and low-risk groups according to the median value of the risk score in all the training, validation and whole groups. Hence, the distribution of risk scores, the correlation between vital status and risk score and the expression pattern of elemental lncRNAs were shown in detail (Figures 2D–F). To investigate the relationship between 8 prognostic lncRNAs and the risk score. Spearman’s correlation analysis was conducted among the expression of element lncRNAs and the risk score (Supplementary Figure S2B). Interestingly, the expression of most members was found strongly correlated with the level of risk score, represented by MIR600HG with a Spearman’s coefficient of −0.54 and CASC8 with 0.58. In addition, certain components of the classifier share a closer relationship in expression. For example, the correlation coefficient between Lnc-PQLC1-10 and CASC8 was more than 0.5, suggesting a potential biological relevance might exist between them in the development of pancreatic cancer.

Afterward, the expression of lncRNAs of the prognostic classifier was compared in groups with different risk levels to further evaluate the differential expression pattern accompanied by the risk score (Supplementary Figure S2C). As a result, 7 out of 8 components (Lnc-ROBO2-3 excluded) of the signature expressed differently between groups with different risk levels. Among them, 5 lncRNAs (Lnc-PQLC1-10, CASC8, Lnc-KAT7-3, MIR924HG and Lnc-PDK2-5) were found up-regulated in patients with a higher risk score, while MIR600HG and Lnc-RAD1-3 expressed lower in the high-risk group. Therefore, more studies and experiments are in great need to explore the possible biological role of these novel lncRNAs in tumorigenesis and the development of pancreatic cancer.

**FIGURE 3:**
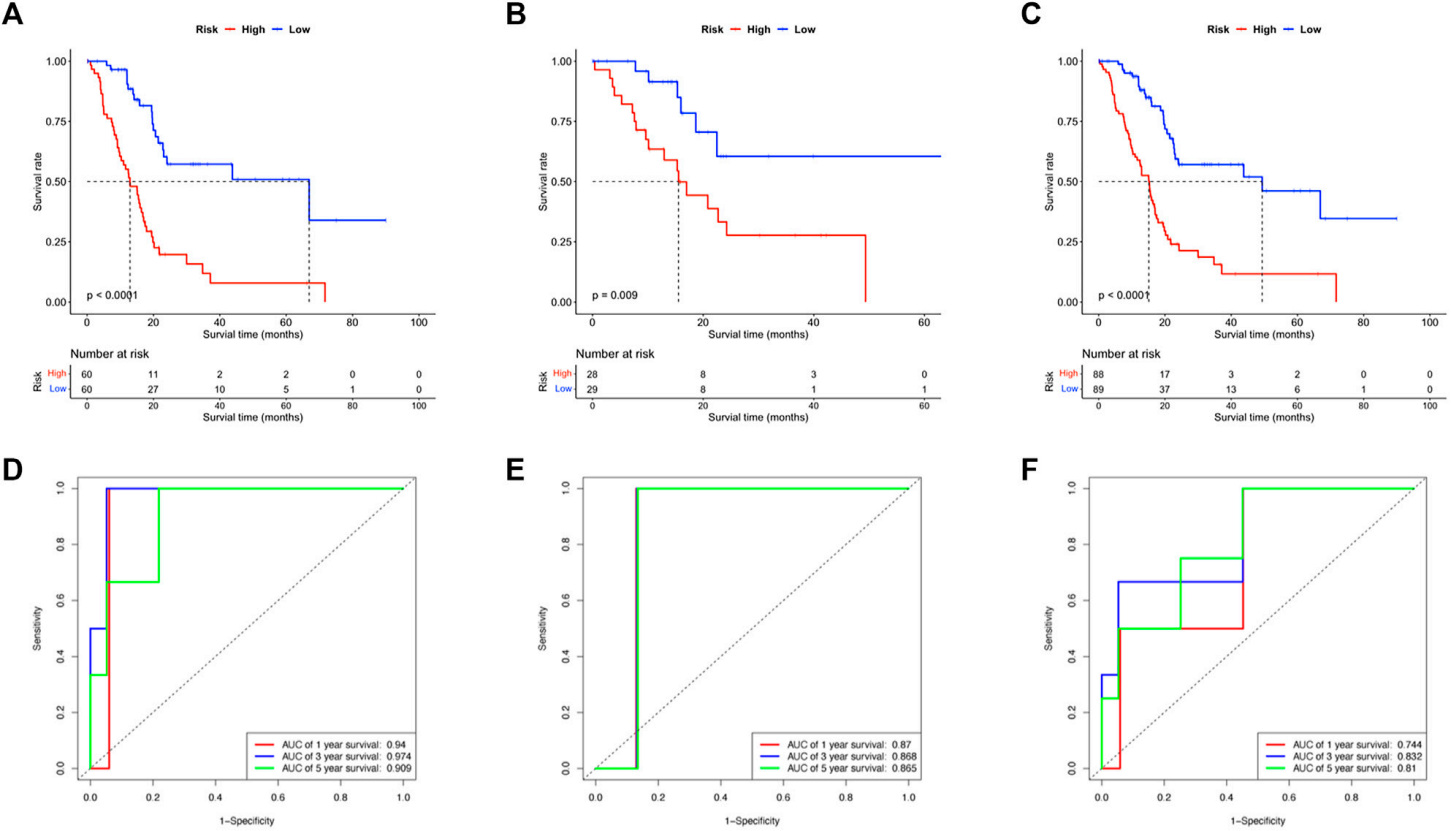
Evaluation of the efficacy of lncRNA based signature. **(A–C)** The Kaplan-Meier survival curves compared the patients in high- and low-risk subgroups of all training, testing and whole cohorts. **(D–F)** The ROC curve anticipated the 1, 3, and 5 years survival of patients in all training, testing and whole cohorts.

For the Kaplan-Meier survival curve, high-risk and low-risk groups were compared in the training, validation, and whole cohorts respectively (Figures 3A–C). In all cohorts, patients in high-risk groups showed significantly poorer outcomes of shorter average survival time where *p* < 0.01 unanimously. These results indicated that the OS- and RFS classifiers are significantly linked with the prognosis of pancreatic cancer, which holds the potential as an effective prediction model. The results claimed that the 8-lncRNA-based signature strongly correlated to the outcome of pancreatic cancer, thus holding the possibility as a prognosis indicator. The time-dependent receiver operating characteristic (ROC) analysis was subsequently performed. The areas under the ROC curve (AUCs) of the classifier were 0.94, 0.97 and 0.90 for 1, 3, and 5 years of anticipation in the training group (Figure 3D), 0.87, 0.86 and 0.86 in the validation group (Figure 3E), 0.75, 0.83 and 0.81 for 1, 3, and 5 years in the whole group (Figure 3F). Moreover, the lncRNA-based panel signature was also assessed when the recurrence happened to predict other outcomes of the disease (Supplementary Figures S3C,D). Despite the signature that could differ the recurrence time and status in K-M regression, it failed to meet comparable accuracy in ROC analysis with less than 0.5 in 5-year anticipation.

### Comprehensive analysis of lncRNA based signature and clinical characteristics

As described, the 8-lncRNAs-based molecular signature was capable of being a novel prognosis indicator with high efficacy for pancreatic cancer. Nevertheless, whether the lncRNA-based signature was relevant to conventional clinicopathologic characteristics remains unclear and requires further study. Clinical data were obtained as previously described and major clinical factors were listed with the risk score for combined analysis.

**FIGURE 4:**
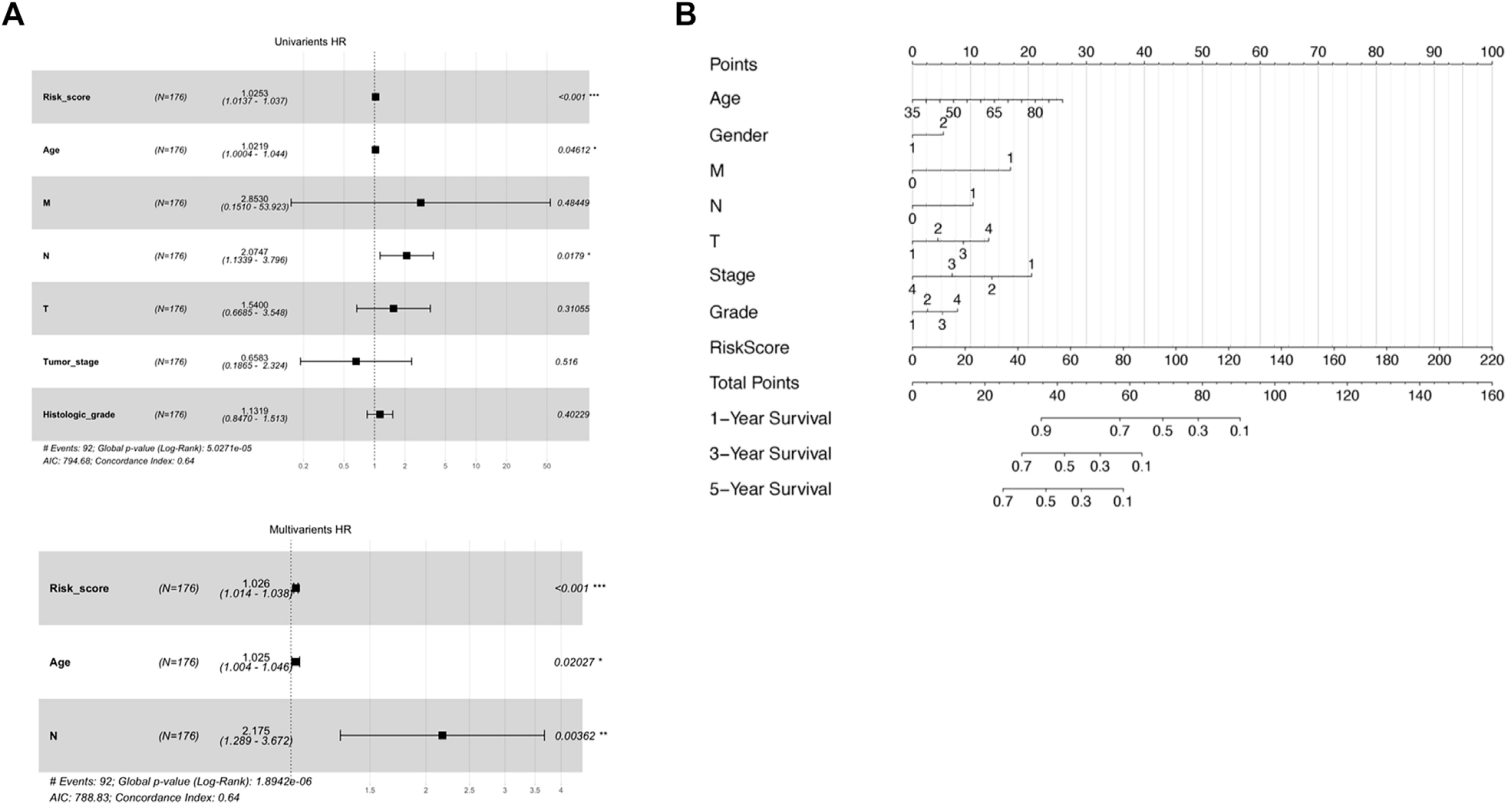
Assessment of prognostic ability of both separate and combined usage of risk score and clinical factors. **(A)** Uni-and multivariate Cox regression by risk score and other clinical factors as OS predictor. **(B)** Nomogram including risk score determined by the lncRNA-based signature and other clinical factors for OS prognostic assessment.

First, the extent of tumor stage and histological grade were found positively associated with the risk score in Pearson’s chi-square analysis, supporting the conclusion that a higher risk score represents advanced tumor progression and worse outcome (Supplementary Figure S3A,B). Next, major clinicopathological characteristics including the risk score were jointly assessed in a two-step Cox regression analysis. As a result, the age of patients, number of malignant lymph nodes, and the risk score were the three factors significantly associated with survival time in a univariate Cox test and thus further went through a multivariate Cox analysis (Figure 4A). Interestingly, all the three variables remained significant in the following multivariate Cox survival test (Figure 4B). Therefore, the 8-lncRNA-based signature and its risk scoring could be considered an independent factor for the prognosis prediction of pancreatic cancer.

To develop a practical, comprehensive model for outcome prediction in pancreatic cancer, a nomogram involving the risk scoring and other clinical characteristics was established (Figure 4C). In the very method, each clinical feature received one certain point according to its statistical weight in prognosis prediction. And the total points reflected the probability of 1, 3, and 5 years of survival. To note, the risk score of the lncRNA-based signature was of most importance and predominance while factors such as gender and histological grade of the tumor weighted minimally in the model.

### Functional enrichment analysis between risk subgroups

**FIGURE 5:**
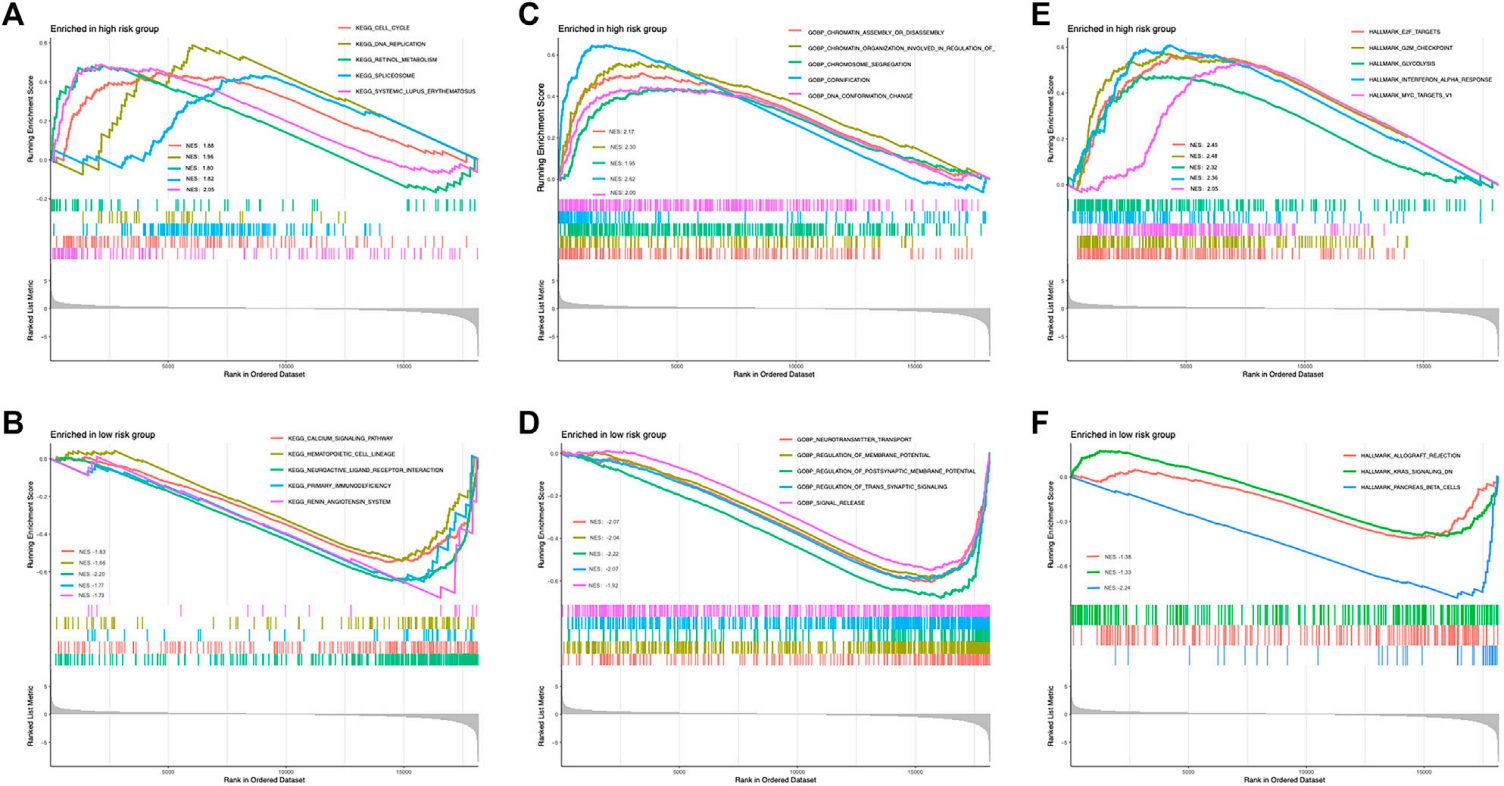
Enrichment of distinguished genes, pathways, and biological processes between low- and high-risk subgroups. **(A)** Top enriched KEGG signaling pathways in high risk group. **(B)** Top enriched KEGG signaling pathways in low risk group. **(C)** Top enriched GO biological processes in high risk group. **(D)** Top enriched GO biological processes in low risk group. **(E)** Top enriched hallmarks in high risk group. **(F)** Top enriched hallmarks in low risk group.

To gain a deeper understanding of the novel mechanism underlying the lncRNA-based molecular classifier, Gene set enrichment analysis (GSEA) was applied to investigate distinguished genes, pathways, and biological processes between subgroups with a different risk scores. In specific, 1755 differentially expressed genes (DEGs) were identified between the high-risk and low-risk subgroups at the condition of absolute log2FC > 1 and *p* < 0.05. For signaling pathways described in Kyoto Encyclopedia of Genes and Genomes (KEGG), these DEGs were significantly enriched in 27 pathways in the high-risk group and 16 pathways in the low-risk group, respectively. Among them, cell cycle, DNA replication, retinol metabolism, spliceosome, and systemic lupus erythematosus were the top 5 KEGG pathways enriched in the high-risk group according to normalized enrichment score (NES), while calcium signaling pathway, hematopoietic cell lineage, neuroactive ligand-receptor interaction primary immunodeficiency and renin-angiotensin system were the most relevant pathways in low-risk group (Figures 5A,B). In addition, biological process (BP) was also evaluated as a major aspect of gene annotation (GO) analysis, where chromatin assembly or disassembly, chromatin organization involved in the regulation of transcription, chromosome segregation, cornification, and DNA conformation change were the most associated BP in the high-risk group, and neurotransmitter transport, regulation of membrane potential, regulation of postsynaptic membrane potential, regulation of trans synaptic signaling and signal release were enriched in the low-risk group (Figures 5C,D).

Last but not least, hallmark gene sets defined by Molecular Signature Database (MSigDB, http://software.broadinstitute.org/gsea/misigdb) were also checked. Interestingly, gene clusters related to E2F targets, G2M checkpoint, glycolysis, interferon α response, and MYC targets were the top hallmarks positively correlated to the high-risk group, but only 3 clusters, allograft rejection, KRAS signaling, and pancreas β cells were relevant to the low-risk group in our whole cohort (Figures 5E,F).

### Estimation of the tumor immune microenvironment

**FIGURE 6:**
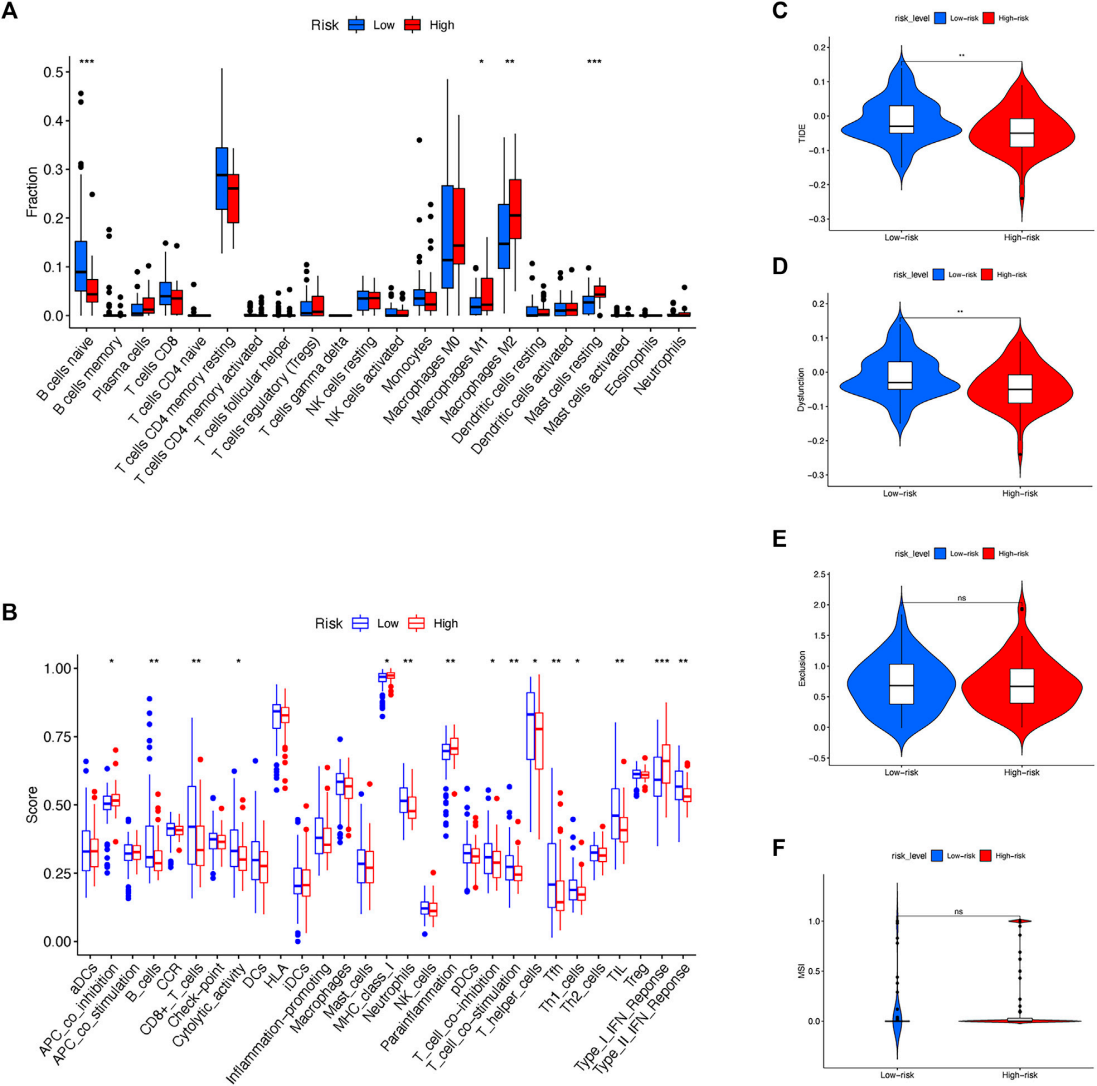
Exploration of immune landscape between low- and high-risk subgroups. **(A)** CIBERSORT algorithm evaluated the level of 22 major immune cells between risk subgroups. **(B)** ssGSEA assessed the extent of main immune cells and activations. **(C)** TIDE scoring was compared between risk sub groups. **(D)** Immune dysfunction was compared between risk sub groups. **(E)** Immune exclusion was compared between risk sub groups. **(F)** MSI was compared between risk sub groups.

With the increasing application of immunotherapy for pancreatic cancer in recent years, it is intriguing to investigate the possible variation of immune microenvironment among risk subgroups. To date, the infiltration and enrichment of 22 main immune cells in the subgroups were analyzed *via* the CIBERSORT algorithm (Figure 6A; Supplementary Figure S4A). The result suggested that patients with a high-risk level tended to have decreased B naïve cells but elevated M1 and M2 subtypes of macrophages and mast cells, while other immune cells remained indifferent. Moreover, Kaplan-Meier survival regression was performed to investigate the effect on patient outcomes imposed by specific immune cells according to CIBERSORT scoring (Supplementary Figure S4B–G). And in agreement with their infiltration profile, B naïve cell, M1/M2 Macrophage and mast cells were also observed to impose a significant effect on the prognosis of patients in the cohort.

Besides CIBERSORT, ssGSEA was also used to compare the activity of some major immune cells and functions between different risk subgroups (Figure 6B). Noticeably, antitumoral immune cells including B cells, CD8^+^ T cells, T helper cells and Neutrophils, accompanied by immune functions such as T cell inhibition, T cell stimulation and type II IFN response were discovered a reduced activation in high-risk rather than low-risk patients.

Since the immune microenvironment was altered among different risk subgroups, it is reasonable to speculate the effect of immunotherapy in risk subgroups might differ simultaneously. Therefore, the expression and risk profile in the cohort were assessed by the Tumor Immune Dysfunction and Exclusion (TIDE, http://tide.dfci.harvard.edu) and compared between risk subgroups (Figures 6C,D). To note, the high-risk group of the cohort received decreasing TIDE score as well as the extent of immune dysfunction, implying a favorable effect of immunotherapy might occur among patients with a high-risk score according to the molecular classifier. Nonetheless, the level of immune exclusion and MSI were equal between high- and low-risk subgroups (Figures 6E,F).

### Identification of potential therapeutic molecules for high-risk score patients

CTRP and PRISM databases, containing large drug sensitivity profiles in thousands of CCLs, are widely used for estimating drug response. CTRP involves 481 compounds from 860 CCLs while PRISM contains 1,448 compounds from 499 CCLs. According to the results above, patients with a high-risk score from our signature were more likely to have a deteriorative outcome. Thus, potential therapeutic agents with higher drug sensitivity were searched particularly for high-risk patients in two different approaches.

**FIGURE 7:**
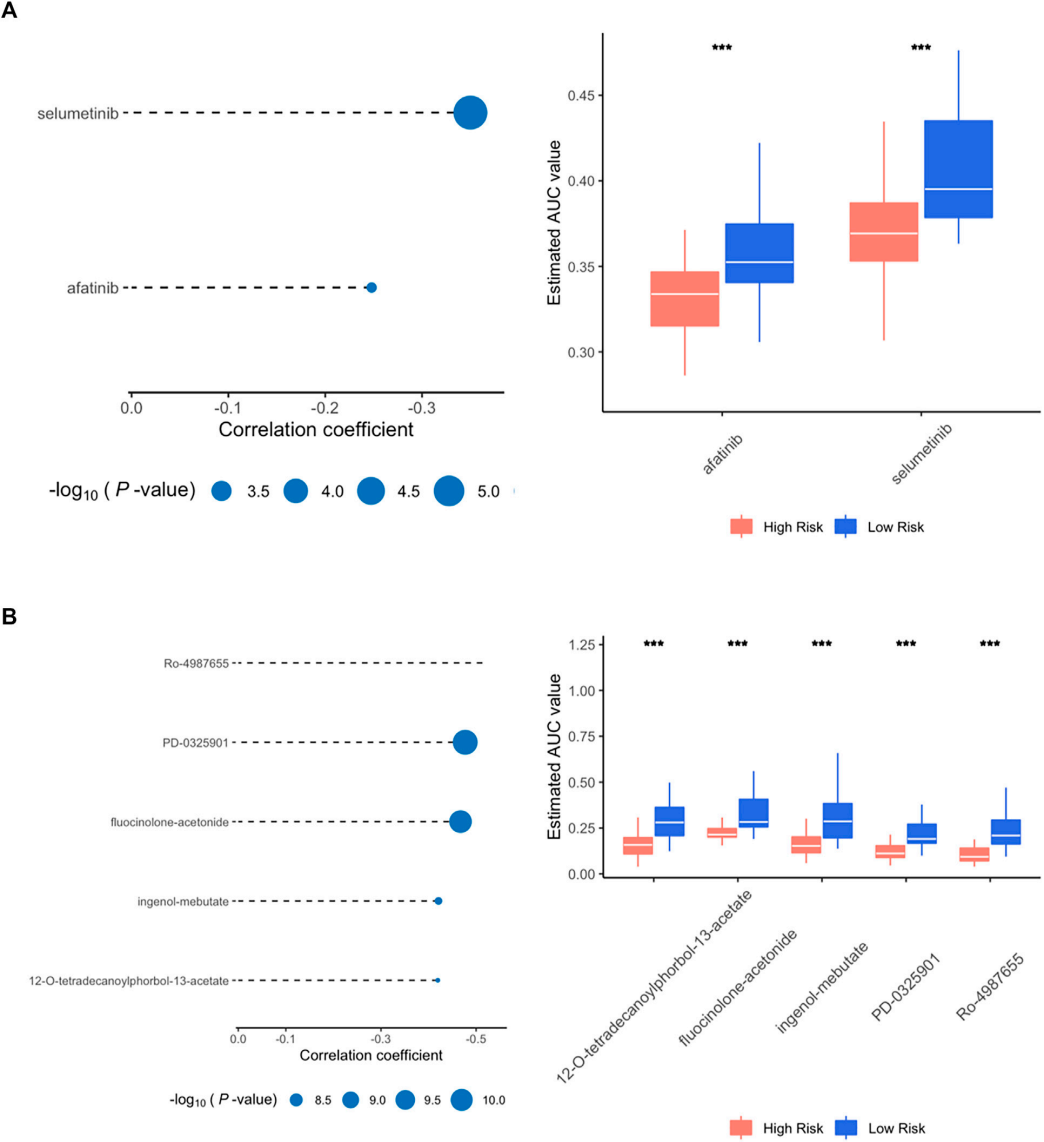
Spearman correlation and differential drug response analysis for high risk group. **(A)** Positive drugs identified in CTRP database. **(B)** Positive drugs identified in PRISM database.

First, drug response profiles were obtained from CTRP and PRISM databases separately. The differential drug response was compared between high- (top 20%) and low-risk (bottom 20%) patients to screen out agents with lower estimated AUC values in high-risk patients (log2FC > 0.05). Subsequently, spearman correlation analysis was applied between the AUC value of specific candidates and the risk score of the patients, in which correlation coefficient *r* < −0.3 for CTRP and *r* < −0.5 for PRISM were considered potentially effective. Altogether, 2 compounds (selumetinib and afatinib) from CTRP and 5 compounds (Ro-4987655, PD-0325901, fluocinolone-acetonide, ingenol-mebutate, 12-O-tetradecanoylphorbol-13-acetate) from PRISM were identified in which all these molecules had a significantly lower value of AUC in high-risk patients compared to low-risk ones (Figures 7A,B).

To further confirm the effectiveness and mechanism of these drug candidates in the cohort, the CMap mode-of-action (MoA) database including nearly 3000 small-molecule compounds was applied. The CMap algorithm compares the expression profile of DElncRNAs in different risk subgroups with the existing response pattern of gene expression for thousands of drugs and molecules in the library. In specific, the CMap score of less than −95 will be considered potentially effective. Positive agents selected *via* CTRP and PRISM were evaluated in the CMap library, respectively. And all agents with CMap score less than −95 were also listed below (Table 2).

**TABLE 2:** Information of candidate agents identified by CTRP, PRISM and CMap databases. Candidate molecules identified by public databases.

Name	Description	Status	CMap score
Selumetinib	MEK inhibitor	Approved	3.28
Afatinib	EGFR inhibitor	Approved	−34.5
Ro-4987655	MEK inhibitor	Phase I	NA
PD-0325901	MEK inhibitor	Phase I/II	2.64
Fluocinolone-acetonide	Glucocorticoid receptor agonist	Approved	0
Ingenol-mebutate	PKC activator	Approved	85.49
12-O-tetradecanoylphorbol-13-acetate	PKC activator	Phase I/II	NA
LY-303511	Casein kinase inhibitor	No data	−98.38
RO-90-7501	Beta amyloid inhibitor	No data	−98.17
TG-101348	FLT3 inhibitor	Phase I/II	−97.64
Baeomycesic-acid	Lipoxygenase inhibitor	No data	−97.24
Pirarubicin	Topoisomerase inhibitor	Phase II	−97.22
PIK-75	DNA protein kinase inhibitor	No data	−96.65

## Discussion

As a lethal malignancy that causes the second most cancer-related death, pancreatic cancer remains a critical global health challenge. Despite tremendous progress has been made during past decades in understanding the genesis and development of this fatal disease, only a fraction of patients have the opportunity to receive radical or surgical resection. Currently, an increasing number of curative approaches including chemotherapy, targeted therapy, or immunotherapy are available for patients with unresectable or metastatic disease, but with merely reluctant effects. To improve the process of clinical decision-making, physicians and surgeons dedicated years to looking for novel strategies for better diagnosing and prognosis guiding for pancreatic cancer. At the moment, clinicians depend largely on pathological factors such as TNM classification, AJCC tumor staging, or histological grade of the tumor to select proper therapy and forecast the outcome for a certain patient. Nonetheless, novel biomarker panels with high accuracy and specificity are widely accepted as a promising approach that could shed light on improving clinical surveillance and management of pancreatic cancer.

In recent years, the roles of non-coding RNA including micro RNA (miRNA), circular RNA (circRNA) and lncRNA have been increasingly emphasized in tumor biology. Numerous studies have revealed that dysregulated lncRNA participates in processes of carcinogenesis and progression of pancreatic cancer. For instance, Liu has reported that lncRNA NR2F1-AS1 promotes proliferation and invasion of pancreatic cancer by regulating the neighboring NR2F1 gene and activating AKT/mTOR signaling pathway (Liu et al., 2022). Huang has demonstrated that lncRNA LNC00842 prompts the malignancy of pancreatic cancer by preventing acetylate PGC-1α from deacetylation and remodeling the metabolic status of cancer cells (Huang et al., 2021). Additionally, Zheng has announced that lncRNA LINC00673 serves as a tumor suppressor by accelerating the ubiquitination of oncogene PTPN11 *via* binding to miRNA-1231 and competing for the endogenous RNA (ceRNA) mechanism (Zheng et al., 2016). As our knowledge of translating molecular profiling and genetic defects into novel biomarkers and targets soars, the potentiality of lncRNA in pancreatic cancer is not to be neglected.

The major pursuit of the present study is to construct a lncRNA-based molecular signature that hosts high efficacy in predicting the outcomes of pancreatic cancer. We have noticed that all patients with pancreatic cancer are now treated with one universal norm mainly based on imaging experiments. The lack of corresponding biomarkers and specified measures possibly result in unsatisfactory therapeutic effects. Therefore, another purpose of the study is to develop a tailored treatment for a certain proportion of patients with possible high risk and worse outcomes.

To achieve these goals, the TCGA-PAAD cohort was randomly split and applied for prognostic analysis. After screening out the differentially expressed lncRNAs and prognostic lncRNAs that were Cox-positive, an RSF model based on minimal depth were established to finally identify target lncRNAs and to form a lncRNA based classifier with the highest efficacy and effectiveness. Hence, the cohorts were subsequentially divided into high-risk and low-risk groups according to the median value of the risk scores by the signature. The survival status of the high-risk and low-risk groups was then compared *via* Kaplan-Meier survival analysis. The result indicates a huge gap between the subgroups where individual samples with high-risk scores reveal remarkably worse prognoses than their counterpart in low-risk groups. In addition, the signature exhibits superior efficacy in prognosis anticipation, where the AUC value in ROC analysis exceeds over 0.90 for all 1, 3, and 5 years prediction of OS in the training group and reaches 0.75 to 0.83 overall. Furthermore, the signature was approved by the uni- and multivariate Cox as one independent predictive factor with marked significance. Last but not least, a combined prediction model that includes multiple associated factors was carried out using a nomogram to put the prognosis prediction of pancreatic cancer into practice.

Despite our analysis suggesting that the lncRNA-based signature is of both expressional and prognostic significance, most component lncRNAs consisting it remains unknown in tumor biology. As the champion with the highest coefficient value among all member lncRNAs, the association between CASC8 and the malignant tumor has been marked recently. It was reported that CASC8 promoted the proliferation of retinoblastoma cells *via* manipulating the methylation of miRNA-34a (Yang B. et al., 2020). Besides, inhibiting CASC8 led to decreased development in non-small cell lung cancer and enhanced sensitivity against chemotherapy, implying CASC8 might be a novel target for cancer treatment in the future (Jiang et al., 2021). MIR600HG is another elemental lncRNA with a high coefficient but rather negative than positive. Pieces of the literature suggested that MIR600HG suppressed metastasis and development by targeting oncogenic ALDH1A3 in colorectal cancer (Yao and Li, 2020). Nevertheless, other studies also indicated that MIR600HG induced but not hindered the progression of the same disease, reflecting its complex nature in tumor biology (Huang et al., 2022). Furthermore, the finding that elemental lncRNAs had considerable mutual correlation in expression suggests some of them might have relevant mechanisms. More functional study is in great need to gain a deeper understanding of these lncRNAs which might unravel novel mechanisms in pancreatic development.

Pancreatic cancer is well known for its feature of immune suppression due to the oncogenic drivers (Bear et al., 2020). By far no single-agent immune therapy was proven clinically effective. And immune modulators are jointly applied with other treatments. After decades of dedication, scientists gradually unraveled the pivotal role of the classical oncogene KRAS and the activation of its mutation in pancreatic cancer. Not only as the trigger of carcinogenesis, but the inception of mutant KRAS signaling also orchestrates a complex network of immunosuppression by manipulating the tumor microenvironment (TME) in pancreatic cancer. Evidence shows that the hyperactivation of KRAS prevents both the innate and adaptive immunity by regulating the expression of immune checkpoint CD47 and PD-L1, activating immune suppressive cells, modulating the level of major histocompatibility complex class I (MHC), forming an inconvenient stromal microenvironment and so forth (cancer cell p2). Despite all the disadvantages, developing novel strategies of immunotherapy for pancreatic cancer is still able to catch the public interest. Several approaches have been proposed for future combinatorial treatments such as stimulating the antigen specificity of T cell immunity, increasing the function of effector T cells, and diminishing the immunosuppressive myeloid and stromal cells.

In this study, the assumption was made that risk subgroups might relate to different immune landscapes. Thus, activation of major immune cells and functions were compared by CIBERSORT and ssGSEA. The result revealed that the activation of a series of major immune cells including B naïve cell, CD8^+^ T cell, T helper cell and neutrophils were modestly down-regulated in the high-risk group, suggesting that the activation of immune cells in tumors of the high-risk patients are possibly paralyzed. Consistently, crucial immune functions such as T cell inhibition, T cell stimulation and IFN response were also found to decline in the high-risk group. Taking together, the enhanced immunosuppression in the high-risk group might be one possible explanation for its notorious outcome.

Of note, as one pivotal component in the tumor microenvironment of pancreatic cancer, the activation of M2 macrophage was found elevated in high-risk patients. The polarization of tumor-associated macrophage (TAM) has been widely accepted as one symbolic event in early and advanced tumorigenesis. Also, several studies confirmed increased M2 deviation of macrophages that promotes tumor behaviors including tumor proliferation, metastasis, and immune escape in pancreatic cancer (Yang S. et al., 2020). Moreover, a meta-analysis containing 1,699 patients with pancreatic cancer concludes that the activity of M2 macrophage is not only closely associated with carcinogenesis, but also has a clear impact on the OS of pancreatic cancer, and thus might be considered a diagnostic and therapeutic target in the future (Yu et al., 2019).

Besides, the gene enrichment and tumor immune analysis unanimously observed an increased interferon-alpha response in the high-risk subgroup. As one of the oldest immune-based therapeutic options for cancer treatment, interferon-alpha is widely used to suppress tumor growth in melanoma, lymphoma, renal carcinoma and so forth. In pancreatic cancer, some clinical trials suggested that chemotherapy based on interferon might improve the overall outcome after surgical resection (Jensen et al., 2014; Ohman et al., 2017). Nonetheless, more efforts are still required to assess the potential of interferon as a first-class approach in a certain population of pancreatic cancer.

In addition, the potential responsibility for the immunotherapy was evaluated by TIDE analysis. Paradoxically, the result indicated that the high-risk group positively correlated with the declined level of TIDE score and immune dysfunction to their low-risk peer, suggesting promising anticipation of therapeutic effect in the high-risk group. To our knowledge, the TIDE algorithm was built on specific tumor types of melanoma and non-small cell lung cancer (NSCLC). The unique characteristic of heterogeneity and immunosuppression in pancreatic cancer could leave the result debatable. In sum, further studies are drastically needed to gain a deeper understanding of immune activity in pancreatic cancer before novel promising therapeutic strategies are to be developed.

Last but not least, potential small molecules that might have therapeutic effects, particularly for high-risk patients were searched *via* CTRP, PRISM and CMap databases. As the only agent identified by CTRP and PRISM with a negative CMap score, Afatinib belongs to the tyrosine kinase inhibitor family and is mainly effective for epidermal growth factor receptor (EGFR) and human epidermal growth factor receptor 2 (HER2). Afatinib, under the commercial name of Gilotrif, has received approval as a first-line treatment for NSCLC. Other indication includes advanced breast cancer with HER2 positive. On-going and complete clinical trials are revealing the potential efficacy of Afatinib in lung cancer other than NSCLC, Head, and Neck squamous cell carcinoma, glioma, and prostate cancer (Molife et al., 2014; Reardon et al., 2015; Hayashi et al., 2022; Kao et al., 2022). Noticeably, two studies exploring the effect of Afatinib on pancreatic cancer acquired only negative results (Haas et al., 2021; van Brummelen et al., 2021). In a phase I study, Afatinib, together with another agent selected from the CTRP database, Selumetinib, was administrated on KRAS-mutated pancreatic cancer (van Brummelen et al., 2021). The result suggested that despite the combination can be used on KRAS-mutated tumors without severe complications, the clinical efficacy was also limited. The other phase II trial concluded that the combination therapy of Afatinib plus gemcitabine did not exhibit a synergistic effect and failed to surpass gemcitabine application alone (Haas et al., 2021). Yet it is still not clear if Afatinib might be more beneficial to certain portions of pancreatic cancer patients.

According to CMap analysis, 5 novel molecules exhibited strong therapeutic potentiality (CMap < −95). LY303511 inhibits the activity of casein kinase 2, which is known to prompt the translation from the G1 to G2 phase and therefore down-regulated cellular proliferation in A549 cells (Kristof et al., 2005). Afterward, LY303511 also increases apoptosis in tumor cells *via* sensitizing TRAIL signaling in HeLa cells (Tucker-Kellogg et al., 2012). However, little is known about the function of LY303511 in pancreatic cancer, and no clinical trial using the agent were carried out so far. TG-101348, a selective JAK antagonist, is one rising star in the antitumor pharmacy. It has been extensively studied in hematology by both *in vitro* and *in vivo* models (Lasho et al., 2008; Wernig et al., 2008; Lasho et al., 2010). Running clinical studies are investigating the role of this promising molecule in leukemia and myeloproliferative neoplasm. Interestingly, a recent study showed that TG-101348 ameliorates hepatic fibro-genesis by inhibiting the TGF-β relied upon activation of hepatic fibroblasts, indicating a broad future prospective of this novel molecule (Akcora et al., 2019).

## Conclusion

To. conclude, this study generated a novel lncRNA-based signature based on a random forest model to predict the overall survival of pancreatic cancer. The efficacy and effectiveness of the signature were evaluated individually and combined with other clinical characteristics. This lncRNA panel, either alone or in combined efforts with other clinical factors, can provide a novel strategy for prognosis anticipation and clinical decision of pancreatic cancer. High-risk patients entitled to the signature tend to have considerably worse outcomes than their low-risk counterparts. And enhanced immunosuppression might be one reasonable explanation. Last but not least, potential therapeutic molecules were excavated from public databases. The result turns out Afatinib, LY-303511, TG-101348 and Pirarubicin could be candidates that are particularly effective for patients with a high-risk score. But before understanding all this, more efforts on validation and mechanistic exploration of these genes and drugs are still in great demand.

## Data Availability

The original contributions presented in the study are included in the article/Supplementary Material, further inquiries can be directed to the corresponding authors.
