# Supplementary material for: Identification of a lncRNA based signature for pancreatic cancer survival to predict immune landscape and potential therapeutic drugs
Source: Front Genet. 2022 Sep 14;13:973444. doi: 10.3389/fgene.2022.973444 (PMC9515791; doi:10.3389/fgene.2022.973444)
Supplement: Supplementary file 1 [file DataSheet1.docx]

**Supplementary figures**


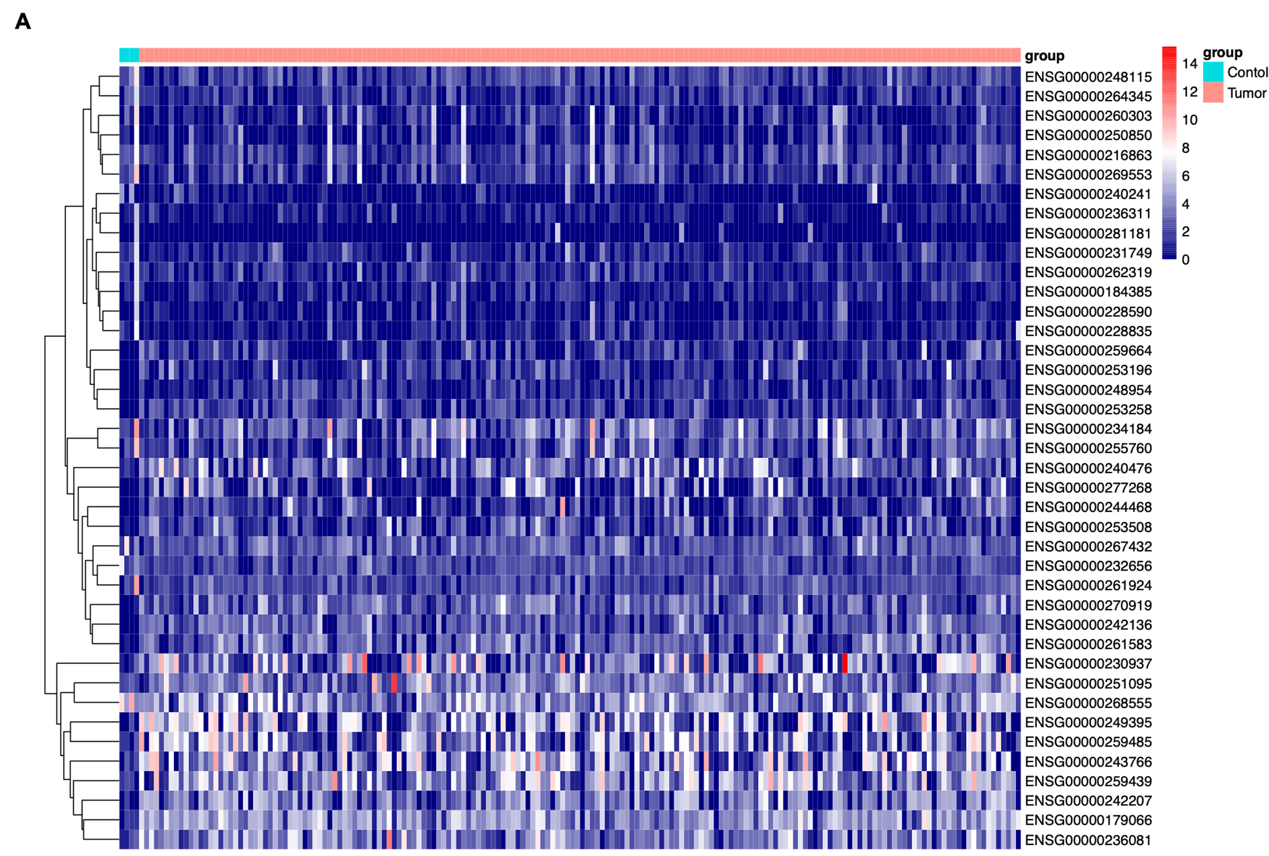


**Supplementary figure 1. Heatmap of differentially expressed lncRNAs in TCGA-PAAD cohort.** A) Heatmap exhibited most up-regulated lncRNAs and down-regulated lncRNAs in TCGA-PAAD cohort (control tissue vs. tumor) by adjust p value.


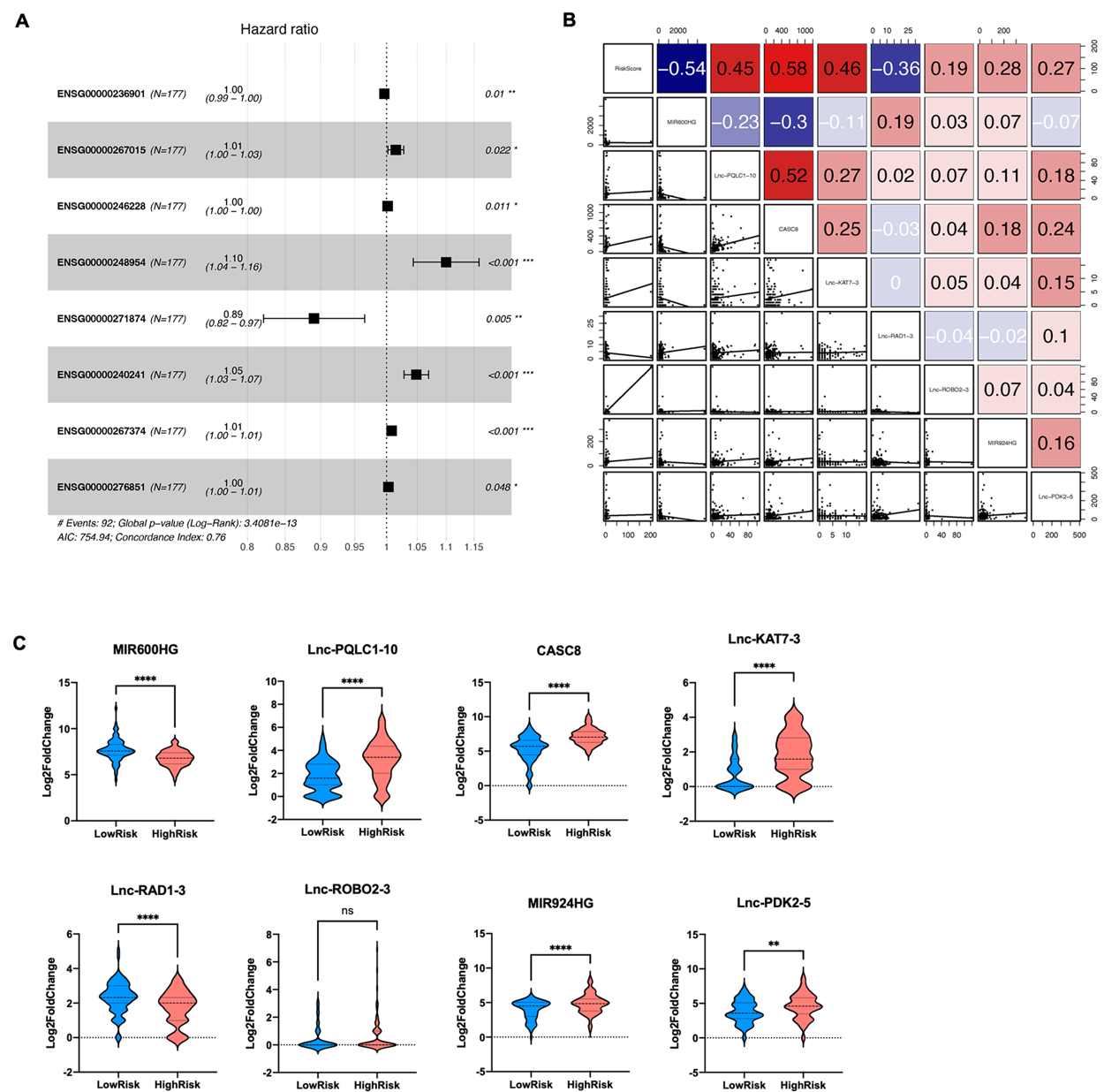


**Supplementary figure 2. The expression feature of the component lncRNAs forming the molecular signature.** A) Cox regression analysis selected candidates from RSF-positive lncRNAs to eventually build the signature. B) Correlation among 8 lncRNAs consisting OS prognosis signature. C) The expression level of lncRNAs consisting OS classifier in low-risk and high-risk groups.


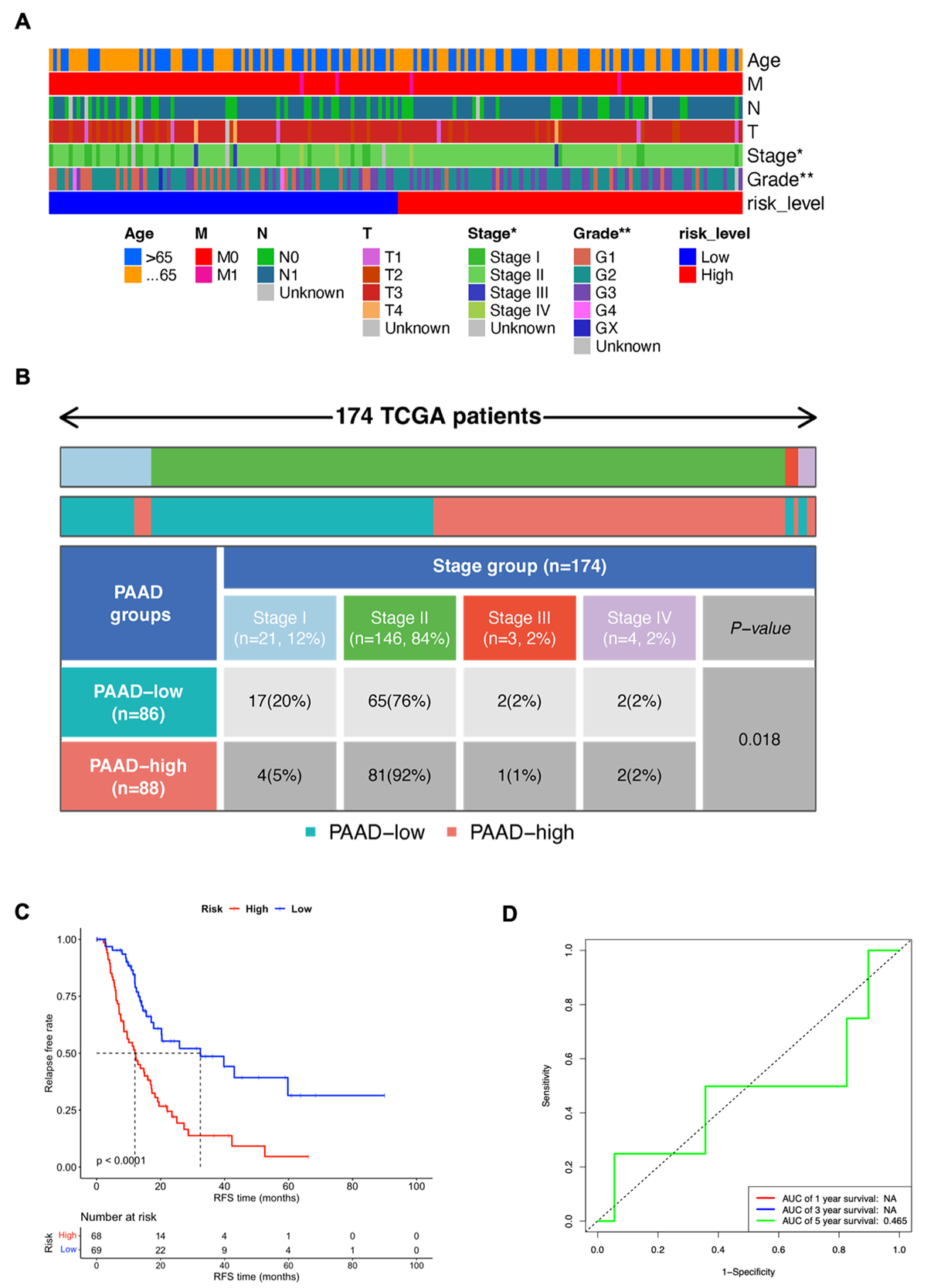


**Supplementary figure 3. Relationship between risk level of the signature and other clinicopathological factors.** A) The correlation of the risk level by the signature were compared with other clinical factors with Pearson’s chi-square analysis. B) The correlation between the risk level and tumor stage was specified and suggested strong significance. C) The Kaplan-Meier survival curves compared the relapse status of patients between high- and low-risk subgroups. D) The ROC curve anticipated the 1, 3, and 5 years relapse of patients.


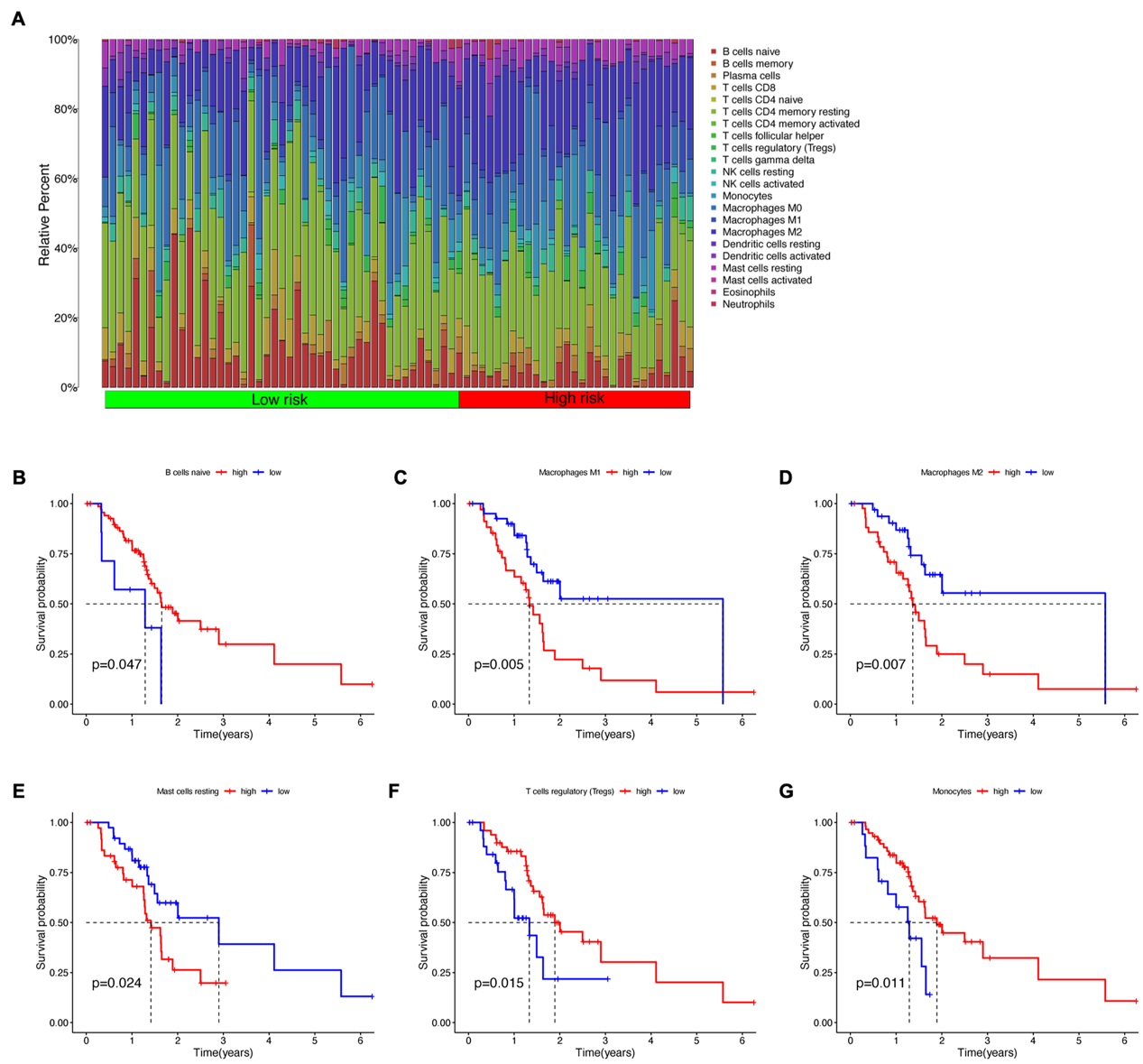


**Supplementary figure 4. Correlation between extent of immune cell activation and survival.** A) Barplot exhibited activation of major immune cells with CIBERSORT analysis. B) Kaplan-Meier analysis showed survival between high- and low-level of B naïve cell. C) Kaplan-Meier analysis showed survival between high- and low-level of macrophage M1. D) Kaplan-Meier analysis showed survival between high- and low-level of macrophage M2. E) Kaplan-Meier analysis showed survival between high- and low-level of mast cell. F) Kaplan-Meier analysis showed survival between high- and low-level of T regulatory cell. G) Kaplan-Meier analysis showed survival between high- and low-level of monocyte.
